# Network analysis reveals crosstalk between autophagy genes and disease genes

**DOI:** 10.1038/srep44391

**Published:** 2017-03-15

**Authors:** Ji-Ye Wang, Wei-Xuan Yao, Yun Wang, Yi-lei Fan, Jian-Bing Wu

**Affiliations:** 1 The Criminal Science and Technology Department, Zhejiang Police College, 555 Binwen Road, Binjiang District, Hangzhou, Zhejiang Province, People’s Republic of China; 2 The department of gastroenterology, The First Affiliated Hospital of Xi’an Jiao Tong University, 277 Yanta West Road, Yanta District, Xi’an, Shanxi Province, People’s Republic of China

## Abstract

Autophagy is a protective and life-sustaining process in which cytoplasmic components are packaged into double-membrane vesicles and targeted to lysosomes for degradation. Accumulating evidence supports that autophagy is associated with several pathological conditions. However, research on the functional cross-links between autophagy and disease genes remains in its early stages. In this study, we constructed a disease-autophagy network (DAN) by integrating known disease genes, known autophagy genes and protein-protein interactions (PPI). Dissecting the topological properties of the DAN suggested that nodes that both autophagy and disease genes (inter-genes), are topologically important in the DAN structure. Next, a core network from the DAN was extracted to analyze the functional links between disease and autophagy genes. The genes in the core network were significantly enriched in multiple disease-related pathways, suggesting that autophagy genes may function in various disease processes. Of 17 disease classes, 11 significantly overlapped with autophagy genes, including cancer diseases, metabolic diseases and hematological diseases, a finding that is supported by the literatures. We also found that autophagy genes have a bridging role in the connections between pairs of disease classes. Altogether, our study provides a better understanding of the molecular mechanisms underlying human diseases and the autophagy process.

Interpreting the biological mechanisms underlying human complex diseases is currently a challenging task in biology and medicine. Until now, great efforts have been devoted to identifying disease-related genes, and this effort has provided resources that aid in understanding the functional processes in diseases. For example, the Online Mendelian Inheritance in Man (OMIM) database is a comprehensive research resource of curated descriptions of human genes and phenotypes and the relationships between them^1^.

Autophagy is a protective and life-sustaining process in which cytoplasmic components are packaged into double-membrane vesicles and targeted to lysosomes for degradation^2^. Accumulating evidence supports the idea that autophagy is involved in many physiological processes including cell metabolism, cell survival, and host defense^3^. It has been reported that autophagy is associated with several pathological conditions, such as cancer and metabolic disease^4^,^5^. Mathias *et al*. found that the role of autophagy in pancreatic ductal adenocarcinoma development is intrinsically connected to the status of the tumor suppressor p53 by a humanized genetically-modified mouse model^6^. Watson *et al*. provided both direct and circumstantial evidence that diminished autophagy flux results in the development of a myelo-proliferative state and accelerates the progress of acute myeloid leukemia (AML) in a mouse model^7^,^8^. Behrends *et al*. reported a proteomic analysis of an autophagy interaction network in human cells under conditions of ongoing (basal) autophagy, providing a global view of the mammalian autophagy interaction landscape^9^. Collectively, these studies suggest the presence of intricate relationships between autophagy processes and different diseases. However, few systematic studies have focused on this hypothesis, and the relationship between diseases and autophagy is not well understood. Thus, a systematic analysis to study the functional links between autophagy and disease is urgently needed.

Recently, several autophagy-related databases have been developed. The Human Autophagy Database^10^ is a public repository containing information about the human genes described so far as involved in autophagy processes. The Autophagy Database^11^ contains basic, up-to-date information on autophagy-related genes and their homologs, covering 41 eukaryotes from the relevant literature. Finally, the Autophagy Regulatory Network^12^ provides an integrated and systems-level database of autophagy, with manually curated, imported and predicted interactions of autophagy components in humans. These resources provide useful information to investigate the functional links between disease and autophagy at the system level. At the same time, with the concept of “-omics” and increasing amounts of high-throughput data, network-based methods are increasingly used to study diseases^13^, and offer a useful tool to study the functional links between disease and autophagy from a systems perspective.

In this study, we focused on the relationships between autophagy processes and diseases. To do this, we constructed a disease-autophagy network (DAN) by integrating known disease genes, known autophagy genes and protein-protein interactions (PPI). Then, we dissected the topological properties of the DAN from a systems level, including degree, clustering and topological coefficient. Next, based on the DAN, a core network was extracted to analyze the functional links between disease and autophagy genes, and we found that autophagy may play an important role in most diseases including cancer and metabolic diseases. We further tested the autophagy genes as serving a bridging role between two pairs of disease classes and used an “intimacy” metric to describe the contribution of autophagy genes in bridging the connections between two pairs of disease classes.

## Results

### The construction of the disease-autophagy network (DAN)

**Figure 1:**
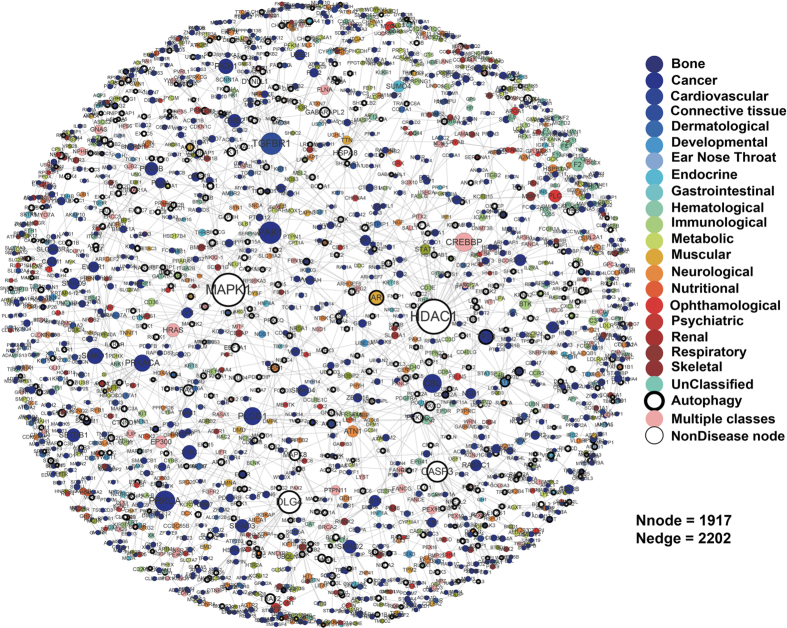
The disease-autophagy network (DAN). The nodes represent genes and the edges represent PPI. Autophagy genes are colored with black borders and disease genes are colored according to their classes. Multiple classes in the figure mean that the genes are involved in multiple disease classes. The node size and font size is proportional to the node degree.

It has been reported that autophagy is associated with several pathological conditions. To explore the functional links between disease and autophagy, we constructed a DAN based on disease and autophagy genes. We mapped 770 autophagy genes and 1317 disease genes to the PPI network of “The Human Protein Reference Database” (HPRD) and then extracted the maximal connected component as the DAN. As shown in Fig. 1, the DAN contained 1917 nodes (disease and autophagy genes) and 2202 edges. Detailed information on the autophagy genes and the classifications of the disease genes is given in the Materials and Methods section.

### Dissection of the DAN

**Figure 2:**
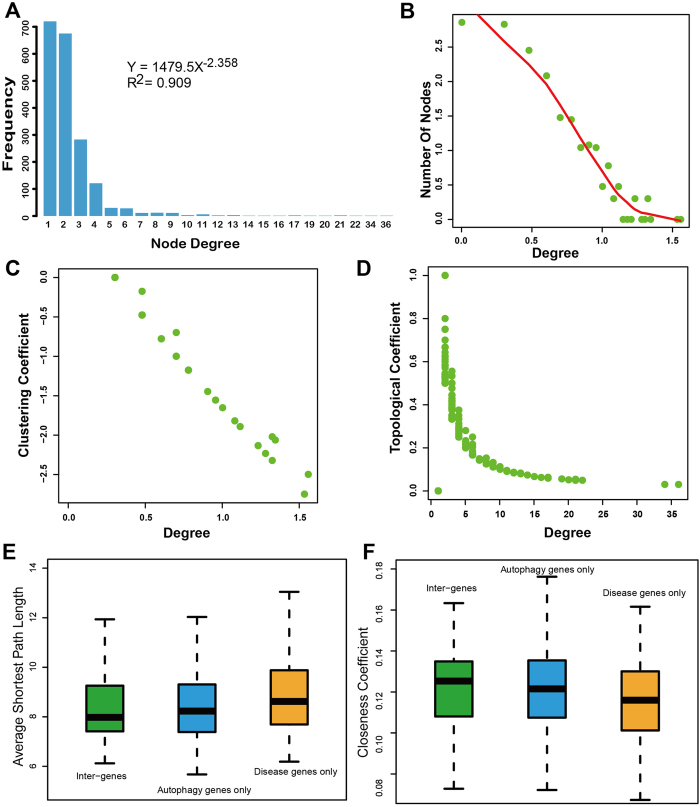
The topological characteristics of the DAN. (**A**) The degree distribution for all the nodes in the DAN is plotted on the x-axis, and the numbers of genes are plotted on the y-axis. **(B)** The degree distribution for all the nodes in the DAN is plotted on the x-axis, and the frequency is plotted on the y-axis. **(C)** The clustering coefficient for all nodes of the DAN. **(D)** The topological coefficients for all nodes of the DAN. The comparison of **(E)** average shortest length and **(F)** closeness coefficient among inter-genes, disease-only genes and autophagy genes.

To dissect the functional cross links in the DAN thoroughly, we examined its topological characteristics, including degree, clustering and topological coefficient. The degree distribution of the DAN followed p(k)∝*k*^−2.356^ as shown in Fig. 2A, which suggests that it is a scale-free network. In scale-free networks, most of the nodes have only small degrees, whereas a few nodes have large degrees (hubs). This property makes the network very robust (in terms of error and the attack tolerance of complex networks). The degree of the DAN spanned from 1 to 32, and most nodes had a degree of 1–4 (Fig. 2B). Next, the clustering coefficient of each node was calculated for the DAN. The clustering coefficient is a measure of the degree to which the nodes in a graph tend to cluster together. As shown in Fig. 2C, we found that the clustering coefficient of the DAN decreased with increasing node degree, suggesting that the DAN is a hierarchical network, which was consistent with previous researches on biological network structures^14^,^15^,^16^. The topological coefficient measures the extent to which a node shares interactions with others in the network. As the degree increased, the topological coefficient decreased (Fig. 2D), suggesting that hub diseases and autophagy genes have fewer common neighbors than others and that the hubs may not be located together in a few densely-connected modules in the DAN.

Autophagy genes seemed to be located in the central of the DAN (Fig. 1). We tested this tendency using the shortest path and closeness coefficients. The closeness coefficient is defined as the reciprocal of the average shortest path length. First, the nodes in the DAN were classified into three types: disease genes only, autophagy genes only, or both disease and autophagy genes (inter-genes). As shown in Fig. 2E, the average shortest path length of the autophagy genes (wilcoxon rank sum test; *P* = 0.00675) and inter-genes (wilcoxon rank sum test; *P* = 0.0081) was much smaller than that of the disease genes. Similarly, the average closeness coefficient of the autophagy genes (wilcoxon rank sum test; *P* = 0.00675) and inter-genes (wilcoxon rank sum test; *P* = 0.0081) was much greater than that of the disease genes (Fig. 2F). These results show that the autophagy genes are much closer and more central than the disease genes.

**Figure 3:**
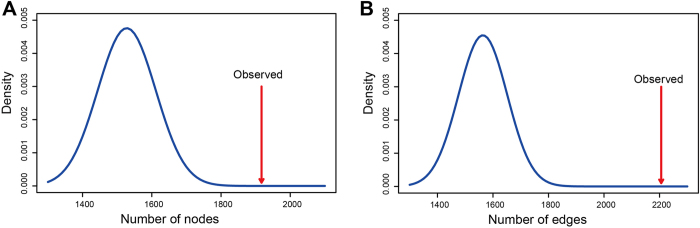
The comparison of the DAN with a randomly generated network. A density plot of the random number of nodes **(A)** and edges **(B)** in 1000 random DANs, with the actual values of the DAN indicated by a red arrow.

To tset the accuracy of the topological analysis, we also compared the DAN with a randomly generated network. First, the edges in the HPRD network were randomly permuted 1000 times with the original degree distributions of the network unchanged. Then, disease and autophagy genes were mapped to 1000 random networks to generate 1000 random DANs. As shown in Fig. 3, the average of the nodes and edges of the 1000 random DANs were much smaller than those of the actual DAN.

### The functional links between disease and autophagy genes

**Figure 4:**
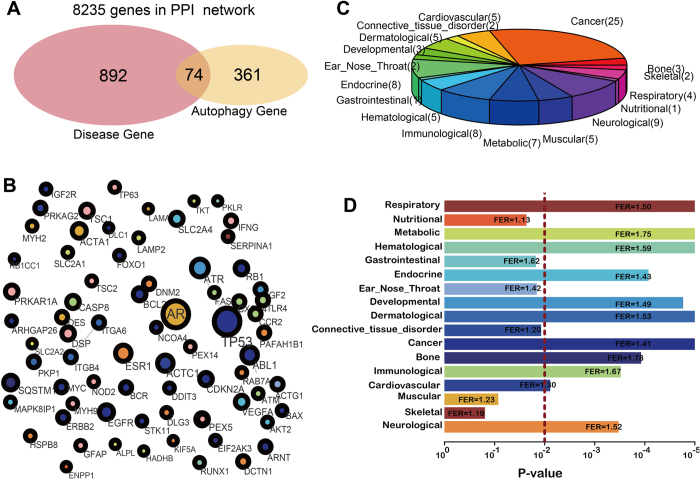
(**A**) The number of overlapping genes between autophagy and diseases in the PPI network. (**B**) The core network generated by mapping inter-genes to PPI network. **(C)** Distribution of inter-genes into different disease classes. **(D)** Fold enrichment ratios (FERs) of overlap between autophagy genes and disease genes in different classes.

To further depict the functional links between disease and autophagy genes, the significance of the overlap between disease genes and autophagy genes with the PPI network as a background was calculated by a hyper-geometric distribution. The observed overlap of 74 genes (inter-genes) was statistically significant (*P* = 1.19 × 10^−8^; Fig. 4A). Then, we mapped these 74 inter-genes to the DAN and extracted a new core network of these genes and their interactions as edges. Interestingly, this new network contained 74 genes but only 4 interactions (Fig. 4B). According to Fig. 2E and F, inter-genes had the shortest paths and the largest closeness when compare to disease genes only or autophagy genes only. This result suggests that these genes may play an intermediate role linking other genes, rather than interacting with each other directly. Next, we classified these 74 genes into different disease classes according to Goh *et al*.^17^. The results showed that these inter-genes were shared by multiple disease classes (Fig. 4C), which suggested that autophagy genes may function in various disease processes. Furthermore, we found that the “cancer” disease class had the most genes (25 genes) that overlapped with autophagy genes, suggesting that autophagy may play an important role in cancer. To test the statistical significance of the overlap between autophagy and disease, fold-enrichment ratios (FERs) and *P*-values were calculated (Fig. 4D). Of 17 disease classes, 11 were significant (wilcoxon rank sum test; *P* < 0.01). The cancer class was the most significantly overlapping with autophagy. It has been reported that autophagy is a highly conserved homeostatic pathway that plays an important role in tumor development and progression by acting on cancer cells via a cell-autonomous mechanism^18^. In addition to cancer classes, metabolic and hematological disease classes were also closely related to autophagy, which has also been supported by previous literature^19^,^20^,^21^. These diseases were termed autophagy-related diseases (ARD), and their related genes are termed ARD genes (ARDG). At the same time, some disease genes showed no overlap with autophagy. The complementary set of ARD were identified as non-autophagy-related diseases (NARD) and their genes as NARD genes (NARDG). To explore the biological functions of the ARDG and NARDG, KEGG pathway enrichment analysis was performed. For the ARDG, these genes were significantly enriched in some cancer-related pathways (such as “pathways in cancer”, “prostate cancer” and “bladder cancer”) as well as pathways highly associated with cancer including the “p53 signaling pathway” and “PI3K-Akt signaling pathway” (Table S1). Conversely, NARDG were enriched in more widely functional pathways such as “complement and coagulation cascades”, “primary immunodeficiency”, and “cytokine-cytokine receptor interaction” (Table S2).

### Autophagy genes as bridges

**Figure 5:**
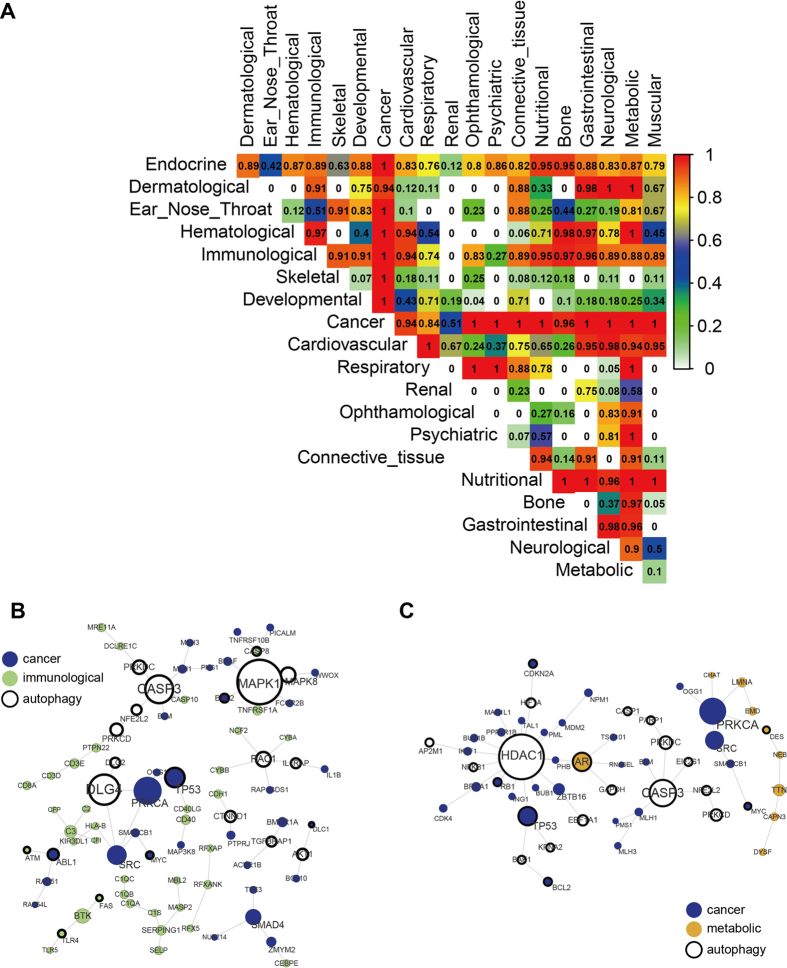
(**A**) The resulting bridgeness of autophagy genes between different diseases classes. (**B,C**) Examples showing the important functions of autophagy genes in connecting disease classes.

These analyses suggested close functional links between certain disease classes and autophagy genes, implying that autophagy genes may play a bridging role between disease classes. To test this, we used an “intimacy” metric to describe the contribution of autophagy genes in bridging the connections between two pairs of disease classes (see Methods). The resulting bridgeness of the autophagy genes between different diseases classes are shown in Fig. 5A. The results showed that autophagy genes play a bridging role between “cancer” and many other classes, for example between “immunological” and “metabolic” classes. We also found that the connections between cancer classes and other diseases bridged by autophagy genes were much stronger than others. This might be due to the close relationship between autophagy genes and cancers; impaired autophagy is closely linked to cancer development and has been extensively studied in a variety of malignancies^22^,^23^,^24^. Other closely connected disease class pairs included “cardiovascular”^25^ and “respiratory”^26^, “Metabolic”^27^ and “Hematological”^28^, and “Nutritional”. Disease pairs between the “immunological” class and many other classes were found to be closely connected by autophagy genes. In the immune system, autophagy is important in the regulation of the innate and adaptive immune responses^3^,^29^. Disruption of these processes, or alterations in the cytokine milieu, can result in inappropriate inflammation characteristic of conditions such as inflammatory bowel disease, cancers, and degeneration^30^,^31^,^32^,^33^.

To show the bridgeness in detail, we focused on several specific diseases. A maximum connected component of a given disease’s genes and some of their connected autophagy genes were extracted from the DAN. We took two pairs of diseases as examples: “cancer” and “immunological” (Fig. 5B) and as well as “metabolic” and “cancer” (Fig. 5C). Generally, genes with higher degrees, such as *MAPK1* (Fig. 5B), *CASP3* (Fig. 5B), *HDAC1* (Fig. 5C) and *PRKCA* (Fig. 5C), tend to be hubs in the gene modules, and are believed to have much more impact on the structure of the network. *MAPK1* (Mitogen Activated Protein Kinase 1) regulates innate immune responses through directly promoting autophagosome formation and lysosomal fusion and regulates cell growth, proliferation, differentiation, migration and apoptosis, playing an important role in cancers and the response to chemotherapeutic agents^30^,^34^. We also noticed that p53 had a higher degree as an inter-gene (Fig. 5B). Studies have indicated that p53 regulation extends to a variety of biological processes including autophagy, fertility, metabolism and immune responses^35^. It can influence the innate immune responses as a tumor suppressor by regulating innate immune TLR genes^36^. Additionally, it has been suggested that there is a direct link between autophagy and cell death via antigen processing, the generation of an inflammatory response and the immune response^37^. Thus, these hub autophagy genes play an important role linking cancers and immunological diseases. In Fig. 5C, *HDAC* (a class I histone deacetylase) was found to have the highest degree. *HDAC* maintains the expression of p53 mutants in human pancreatic cancer cells and is considered a potential anticancer target^38^. Additionally, *AR* (androgen receptor) was found to connect cancers and muscular diseases as an inter-gene. Suppression of AR transcriptional activity and gene expression was found to activate autophagy in prostate cancer (PC)^39^ and targeting AR may induce PCa cell apoptosis, known as autophagic cell death^40^. It has been reported that dysregulation of androgens or AR signaling perturbs normal reproductive development and accounts for a wide range of pathological conditions such as androgen-insensitive syndrome, PC, and spinal bulbar muscular atrophy^41^. Further studies on these genes are still needed to understand the underlying cellular and molecular mechanisms of the roles of autophagy genes in linking different kinds of diseases.

## Discussion

Autophagy is a constitutive lysosomal catabolic pathway that degrades damaged organelles and protein aggregates. Dysregulation of autophgosome formation and autophagy flux can have deleterious consequences, ranging from a failure to “clean house” to the induction of autophagy-induced cell death. It has been widely accepted that autophagy is associated with several pathological conditions. However, systematic analyses of the functional links between autophagy and disease remain in their early stages.

Here, we constructed a DAN by integrating known disease genes, known autophagy genes and PPIs. Then, we dissected the topological properties of the DAN, including degree, clustering and topological coefficient. These properties uncovered that the DAN is a scale-free network. By analyzing the closeness, autophagy genes and inter-genes were closer and more central than disease genes, suggesting that autophagy genes are important in the maintenance of the network structure. Next, a core network from the DAN was extracted to analyze the functional links between disease and autophagy genes. The genes in this core network were significantly enriched in multiple disease related pathways, showing that autophagy genes may function in various disease processes. To test the statistical significance of the overlap between autophagy and disease, FERs and *P*-values were calculated. Of 17 disease classes, 11 were significant, including cancer, metabolic diseases and hematological disease, which are supported by literature. Pathway enrichment analysis of the ARDG and NARDG showed that they are involved in different biological processes. To further inspect the bridging role autophagy plays in diseases, we used an “intimacy” metric to evaluate the contribution of autophagy genes in bridging the connections between two pairs of disease classes. The results showed that autophagy genes served as a bridging role linking different disease classes, such as cancer and immunological, as well as cancer and muscular diseases.

To show the robustness of the conclusion, we used the largest human curated signaling network^42^ (HCSN; http://www.cancer-systemsbiology.org/data-software) to re-build the DAN network and then re-ran the analysis (detailed information in Supplementary Text). The results showed that the new network (DAN_HCSN) had similar topological characteristics (degree, clustering and topological coefficient) with DAN, and autophagy genes also show much closer and more central than disease genes in the DAN_HCSN (Figures S1 and S2). Furthermore, similar to previous results, the DAN_HCSN also revealed close connections between “cancer” and many other disease classes by autophagy genes (Figures S3 and S4).

We also notice that our analysis relied on the topology of the network; therefore bias may have been introduced by the incompleteness of data. First, the incompleteness of the PPI data may limit the analysis. Additionally, the disease and autophagy genes from databases may have higher degrees simply because they are better studied This situation will improved as more accurate and complete resources became available. Despite the potential presence of bias, our study developed a systems-level method to uncover the functional links between diseases and autophagy, which may provide a better understanding of the molecular mechanisms underlying human diseases and autophagy processes.

## Materials and Methods

### Datasets

Autophagy genes were curated from three sources. The Human Autophagy Database (http://www.autophagy.lu/)^10^, which provides a complete and an up-to-date list of human genes and proteins involved directly or indirectly in autophagy as described in the literature; the Autophagy Database (http://tp-apg.genes.nig.ac.jp/autophagy)^11^, provides basic, up-to-date autophagy-related genes and their homologs in 41 eukaryotes from the relevant literature; and the Autophagy Regulatory Network (http://autophagy-regulation.org)^12^ contains manually curated, imported, and predicted interactions of autophagy components in humans. By combining the three resources, we obtained 770 autophagy genes.

Disease genes were obtained from OMIM, and their class information was extracted from Goh *et al*.^17^. The diseases in this study were manually classified into 20 disease classes based on the physiological system affected. Diseases with multiple clinical features were assigned to the “multiple” class and diseases with no clear classes were defined as “unclassified”. Finally, 1317 disease genes sorted into 20 disease classes were obtained.

The PPI network was downloaded from the HPRD database^43^. In total, there were 8326 proteins with 31368 interactions.

### Topological measurement

For a given graph G = (V, E), in which V represents a set of nodes, and E represents a set of edges. Degree is a measurement of how many edges link to the nodes and it reflects the interactions of this node with other nodes. If there are k edges linked to node v, then degree D of node v is defined as:


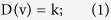


The clustering coefficient measures how close the neighbors of a node are connected. It is defined by the proportion of links between the nodes within its neighbors divided by the number of links that could possibly exist between them. Then the clustering coefficient can be calculated as:


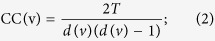


where T represents the number of triangles incident to node v, and *d(v*) denotes the degreeof node v.

The topological coefficient measures the extent to which a node shares interactions with others in the network, which can be defined as:


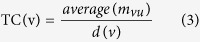


where *m*_*vu*_ represents the number of common nodes between v and u, and *d(v*) denotes the degreeof node v.

The closeness coefficient represents how close a node is to other nodes in the same network and is defined as the average mean path from one node to all other nodes. The closeness coefficient (C) of node v is defined as:


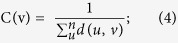


where d(u, v) denotes the shortest distance between node u and node v, n denotes the number of nodes in the network.

### The significance of overlapping genes

The significance of the overlap between autophagy genes and disease genes against the nodes of the PPI network was calculated by a hyper-geometric distribution. Considering that a set of N elements has two subsets with m and n elements respectively, the probability of containing at least x overlapping elements is derived with the following formula:


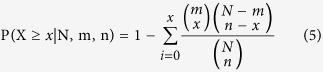


### Fold-enrichment ratio (FER)

The FER is defined as the ratio between the observed value and expected value, which is described as follows:


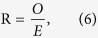


where O is the observed value and E is the expected value.

### Intimacy between each disease pair

The “intimacy” measure is used to describe the contribution of autophagy genes in bridging the connections between pairs of disease classes. Given a pair of diseases *d*_*i*_ and *d*_*j*_ with corresponding disease gene sets {*g*_i1_, *g*_i2_, …. *g*_*km*_} and {*g*_*j*1_, *g*_*j*2_, …. *g*_*jm*_}, considering the disease information passed from *d*_*i*_ to *d*_*j*_ on the DAG, the connections between *D*_*j*_ and *D*_*i*_ are defined as


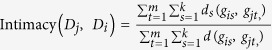
, in which *d*_*s*_(*g*_*is*_, *g*_*jt*_) represents the number of shortest paths between *g*_*is*_ and *g*_*it*_. *d*_*s*_(*g*_*is*_, *g*_*jt*_) = *d*_*s*_(*g*_*is*_, *g*_*jt*_) when the autophagy genes exist in the shortest path, else *d*_*s*_(*g*_*is*_, *g*_*jt*_) = 0.

### Enrichment analysis

KEGG and GO functional enrichment analysis was performed using DAVID (https://david.ncifcrf.gov/). It provides a useful and comprehensive set of functional annotation and enrichment tools for researchers to understand the biological mechanisms of a gene set of interest^44^.

## Additional Information

**How to cite this article**: Wang, J.-Y. *et al*. Network analysis reveals crosstalk between autophagy genes and disease genes. *Sci. Rep.* **7**, 44391; doi: 10.1038/srep44391 (2017).

**Publisher's note:** Springer Nature remains neutral with regard to jurisdictional claims in published maps and institutional affiliations.

## Supplementary Material

Supplementary Information

Supplementary Table S1

Supplementary Table S2

Supplementary Table S3

Supplementary Table S4
