# Supplementary material for: Network analysis reveals crosstalk between autophagy genes and disease genes
Source: Sci Rep. 2017 Mar 15;7:44391. doi: 10.1038/srep44391 (PMC5353691; doi:10.1038/srep44391)
Supplement: Supplementary Information [file srep44391-s1.pdf]

Network analysis reveals crosstalk between autophagy genes and disease genes

Ji-Ye WANG[1*],Wei-Xuan YAO[1], Yun WANG[2], Yi-lei FAN [1], Jian-Bing WU [1]

[1] The Criminal Science and Technology Department, Zhejiang Police College, 555 Binwen Road, Binjiang District, Hangzhou ,Zhejiang Province, People's Republic of China; [2] The department of gastroenterology, The First Affiliated Hospital of Xi'an Jiao Tong University, 277 Yanta West Road, Yanta District, Xi’an, Shanxi Province, People's Republic of China ;

Running title: Network analysis reveals crosstalk between genes and disease by autophagy

Address correspondence to: Ji-Ye WANG, Zhejiang Police College, 555 Binwen Road, Binjiang District, Hangzhou ,Zhejiang Province, People's Republic of China;. Tel: 86-0379 87787029; Fax: 86-0379 87787029; E-mail: Wangjiye_poli@163.com

Disclosure of conflict of interest

The authors declare that there is no conflict of interests regarding the publication of this paper.

We downloaded the largest human curated signaling network (HCSN) (http://www.cancer-systemsbiology.org/data-software). It contained 6904 genes and 62,937 interactions.

First, similar to a previous DAN, we constructed a second network based on mapping disease and autophagy genes to the HCSN. Then, we extracted the maximal connected component as the DAN_HCSN. The DAN_HCSN contained 1086 nodes (disease genes and autophagy genes), with 5981 edges.

To analyze the DAN_HCSN, we also examined its topological characteristics, including degree, clustering and topological coefficient. Similar to previous results, the degree distribution of DAN_HCSN also followed a power-law distribution, $Y = 448x^{-1.393}$ (R square = 0.897), suggesting that the DAN_HCSN has a scale-free feature (Figures S1A and S1B). Also, the clustering coefficient of the DAN_HCSN decreased as the node degree increased, suggesting that it is a hierarchical network (Figure S1C). Additionally, with increasing degree, the topological coefficient decreased in the DAN_HCSN (Figure S1D). The shortest path and closeness coefficient were also measured. As shown in Figure S1E, the average shortest path length of the autophagy genes (P = 0.0031) and inter-genes ($P = 3.16 \times 10^{-7}$) was much smaller than that of the disease genes (Figure S2E). Similarly, the average closeness coefficient of the autophagy genes (P = 0.0031) and inter-genes ($P = 3.16 \times 10^{-7}$) was much greater than that of the disease genes (Figure S2F). These results support the observation that autophagy genes were closer and more central than the disease genes in the DAN_HCSN. Similarly, we also compared the DAN_HCSN with randomly generated networks. First, the edges in the HCSN network were randomly permuted 1000 times with the original degree distributions of the network unchanged. Then, disease and autophagy genes were mapped to 1000 random networks to generate 1000 random DAN_HCSNs. As shown in Figure S2, the average number of nodes and edges of the 1000 random DAN_HCSNs was much smaller than that of the real DAN_HCSN.

To further depict the functional links between disease and autophagy genes in the DAN_HCSN, the significance of the overlap between disease and autophagy genes

within the PPI network as a background was calculated by a hyper-geometric distribution. With an overlap of 67 genes (inter-genes), significant overlap was observed $P = 0.006$ (Figure S3A). The results showed that these inter-genes also belonged to different disease classes (Figure S4C). Similar to previous results, we found that the “cancer” disease class had the most genes (18 genes) overlapping with autophagy genes, suggesting that autophagy may play an important role in cancer. To test the statistical significance of the overlap between autophagy and diseases, the FER and *P*-values were calculated (Figure S4D). Of 17 disease classes, 10 were significant ($P < 0.01$). Not surprisingly, the “cancer” class was the most significantly overlapping with autophagy. To explore the biological function of the ARDG and NARDG in the DAN_HCSN, KEGG pathway enrichment analysis was performed. Similar to previous results, ARDG was also significantly enriched in some cancer-related pathways (such as “pathways in cancer,” “prostate cancer,” and “bladder cancer”) and pathways highly associated with cancer including “p53 signaling pathway” and “PI3K-Akt signaling pathway” (Table S3). Meanwhile, NARDG was enriched in more broad functional pathways such as “complement and coagulation cascades,” “primary immunodeficiency” and “cytokine-cytokine receptor interactions” (Table S4).

To test the bridging role of autophagy genes, we used the “intimacy” metric to describe the contribution of autophagy genes in bridging the connections between pairs of disease classes in the DAN_HCSN. The resulting bridgeness of autophagy genes between different diseases classes is shown in Figure S4. The results showed that there are close connections between different disease classes by the autophagy genes, for example between the “Immunological” and “Metabolic” classes. We also found strong connections between cancer classes and other diseases. This might be due to the close relationship between autophagy genes and cancer.

In summary, the results of the two networks were robust.

Figure S1

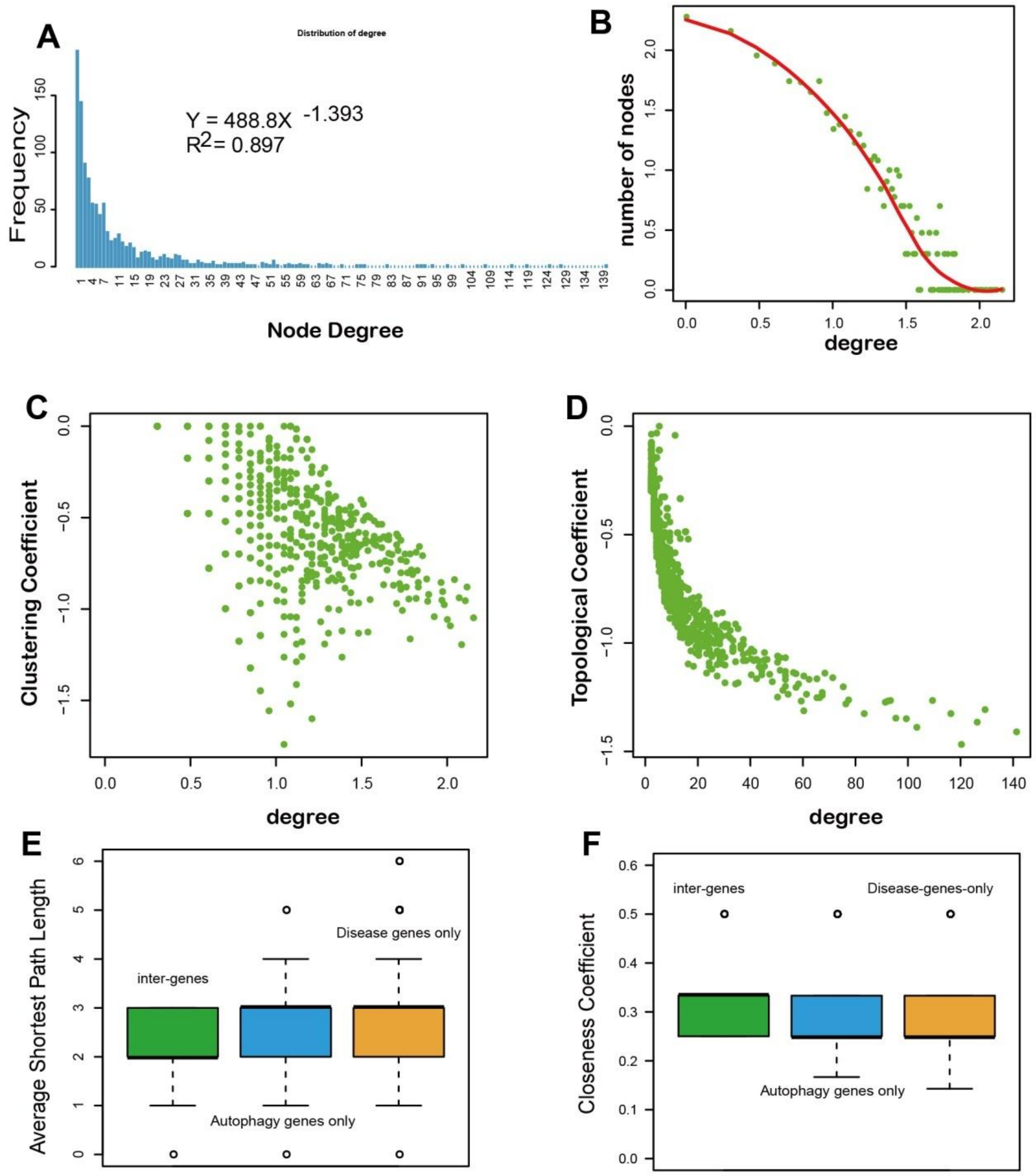

Figure S1. The topological characteristics of the DAN_HCSN. **(A)** The degree distribution for all the nodes in the DAN_HCSN is plotted on the x-axis, and the numbers of genes are plotted on the y-axis. **(B)** The degree distribution for all the nodes in the DAN_HCSN is plotted on the x-axis, and the frequency is plotted on the y-axis. **(C)** The clustering coefficient for all nodes of the DAN_HCSN. **(D)** The topological coefficients for all nodes of the DAN_HCSN. The comparison of **(E)** average shortest length and **(F)** closeness coefficient among inter-genes, disease-only genes and autophagy genes

Figure S2

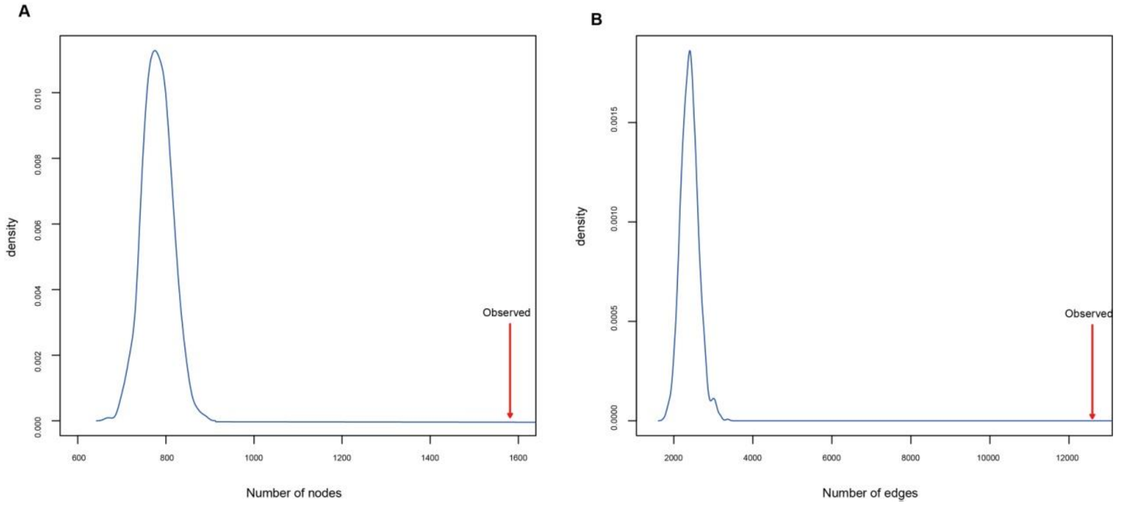

Figure S2. The comparison of the DAN_HCSN with a randomly generated network. A density plot of the random number of nodes **(A)** and edges **(B)** in 1000 random DAN_HCSNs, with the actual values of the DAN_HCSN indicated by a red arrow.

Figure S3

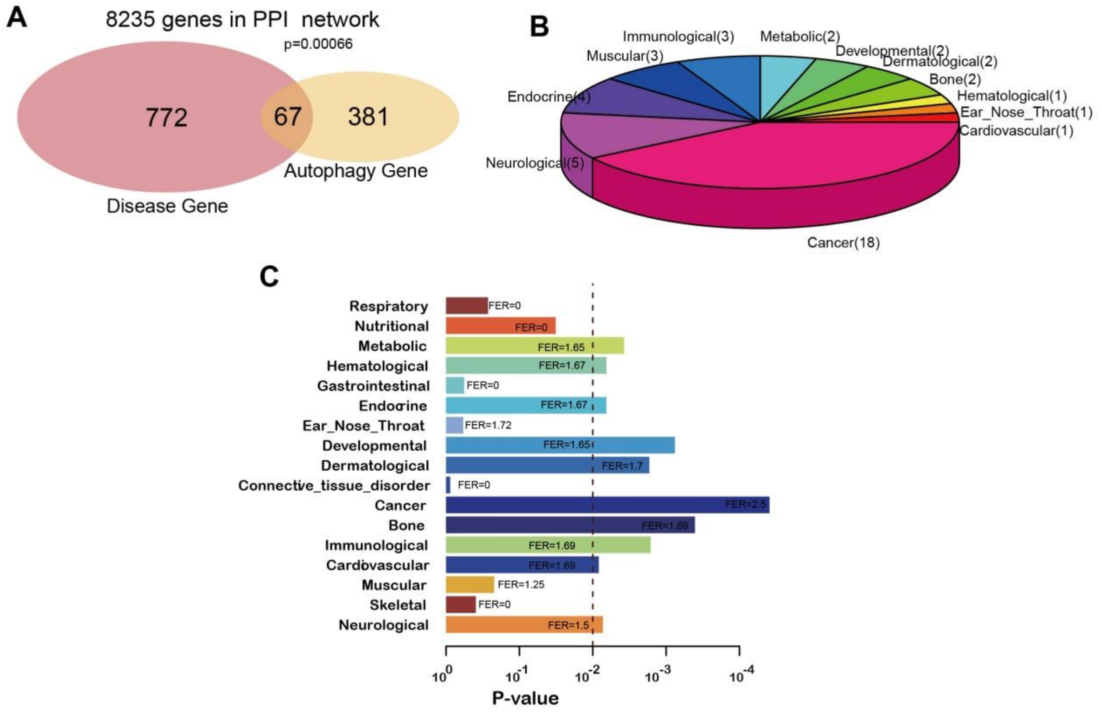

Figure S3. (A) The number of overlapping genes between autophagy and diseases in the PPI network. **(B)** Distribution of inter-genes into different disease classes. **(C)** Fold enrichment ratios (FERs) of overlap between autophagy genes and disease genes in different classes.

Figure S4

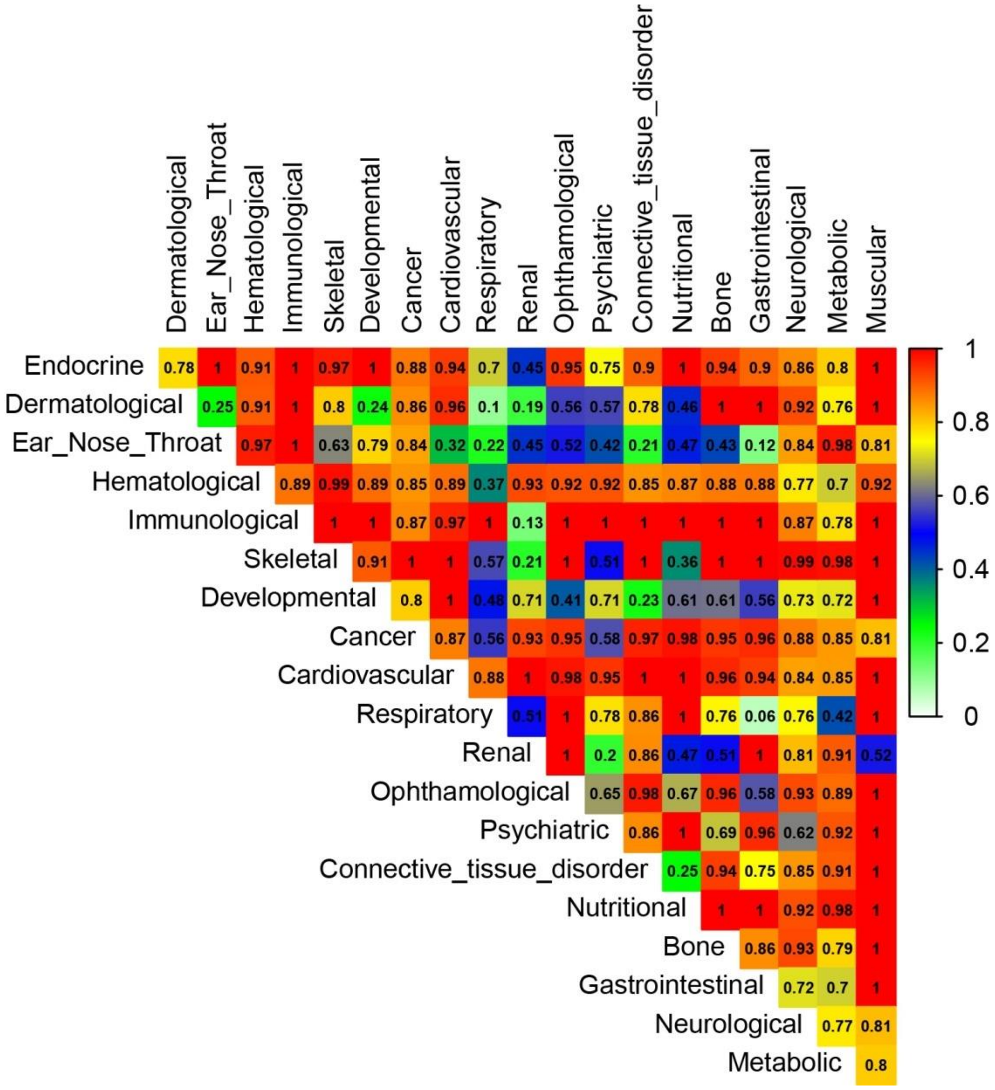

Figure S4. The resulting bridgeness of autophagy genes between different diseases classes.

Table S1. KEGG pathway enrichment analysis of the ARDG.

Table S2. KEGG pathway enrichment analysis of the NARDG.

Table S3 KEGG pathway enrichment analysis of the ARDG of DAN_HCSN

Table S4 KEGG pathway enrichment analysis of the NARDG of DAN_HCSN
